# Influencing the body schema through the feeling of satiety

**DOI:** 10.1038/s41598-022-06331-3

**Published:** 2022-02-11

**Authors:** Patricia Baumann, Nina Beckmann, Stephan Herpertz, Jörg Trojan, Martin Diers

**Affiliations:** Department of Psychosomatic Medicine and Psychotherapy, LWL University Hospital, Ruhr University Bochum, Alexandrinenstraße 1-3, 44791 Bochum, Germany

**Keywords:** Psychology, Human behaviour

## Abstract

The body schema is a much discussed aspect of body awareness. Although there is still no single definition, there is widespread consensus that the body schema is responsible for movement and interaction with the environment. It usually remains outside of active consciousness. There are only few investigations on influences on the body schema and none of them investigated feeling of satiety or hunger. Thirty-two healthy women were investigated twice, one time sat and the other time hungry. To measure the body schema, we used a door-like-aperture and compared the critical aperture-to-shoulder-ratio (cA/S). A cover story was used to ensure that the unconscious body schema has been measured. We found a significantly higher cA/S for satiety compared to hungry, which indicates that during satiety participants rotate their shoulders for relatively larger door compared to hunger, unconsciously estimating their body size to be larger. We showed that even a moderate rated feeling of hunger or satiety leads to an adjustment in body-scaled action and consequently also an adaptation of body schema. It suggests that, in addition to the visual-spatial and the proprioceptive representation, somatic information can also be relevant for the body schema.

## Introduction

There are a lot of concepts and definitions about body perception. Body experience is often called the generic term of body perception and is divided in different subclasses^1^. A frequent division is the distinction between the conscious body image and the unconscious body schema^2–4^. The body image combines the cognitive, emotional and perceptual awareness of one’s own body^3,5,6^. The body schema is the sensorimotoric representation of the body^5^ and is responsible for automatically performed motions and the interaction of the body with its environment, e.g. integrating body postures in the environmental space^2,3,7–10^.

In interaction with the environment visual-spatial and proprioceptive perception play a particularly important role for the body schema. To investigate these aspects, full body ownership illusions, also in virtual reality (VR), have been used. The more lifelike the virtual body looks, the better the illusion works^11^. If the virtual body of the illusion is seen from the perspective of the first and not the third person, a visual congruence is sufficient for the illusion to function^11,12^. Tactile stimulations are felt more real within such illusions if they are synchronized on the own and on the virtual body^13^. During congruent stimulation the illusion was perceived as more spatial and leads to a better adaptation to the virtual body while moving^14^.

In addition to exteroceptive perception, aspects of interoception can also influence body perception. Interoception is the perception of the state of the body. It refers to the ability to sense the own physiological conditions such as temperature, pain, itch, respiration, satiety or heartbeat^15,16^. One potential method of assessment of the body schema is the laterality judgment task (LJT). In this task hands in different orientations are presented to the participants, which have to decide whether it is a right or a left hand^16^. Patients with various chronic pain syndromes such as chronical musculoskeletal pain or complex regional pain syndrome^17,18^ achieved poorer results in the LJT than healthy subjects. These results suggests that the pain perception as part of the interoception is one variable among others that has an influence on the body schema, nevertheless the interoceptive sensitivity was not directly measured.

Another interoceptive input is the feeling of hunger and satiety. Various brain regions (e.g. hypothalamus, inferior parietal lobe (IPL) or the brain stem), hormones (e.g. leptin and ghrelin) and different cell types (e.g. Langerhans’ cells in pancreas or nerve cells of the vagus nerve) are involved in the regulation of hunger and satiety^19–22^.

So far, there is not much research about the effect of satiety or hunger on body perception. In particular, the relationship between the level of saturation and the body schema has not yet been investigated. However, for the body image a positive association between gastric interoception and aspects of the body image (e.g. body appreciation and functionality appreciation) could be found^23,24^. Additionally, food intake influences the body image. Subjects who drank a milkshake during the experiment showed greater dissatisfaction with their body and a greater discrepancy towards their ideal body image than subjects who did not drink a milkshake^25^.

Satiety has also an influence on the evaluation of the attractiveness of other persons. Men who were hungry found women with a higher BMI more attractive than when they felt satiety^26^ and additional to larger bodies also larger objects were found more attractive by hungry participants^27^.

Both, exteroceptive and interoceptive influencing factors are known for the modulation of the body schema. So far there are no studies on the latter that have dealt with the influence of satiety. The aim of the study was to investigate a possible influence of the feeling of satiety or hunger as interoceptive factors on the body schema. We hypothesis that the feeling of satiety leads to a greater critical aperture-to-shoulder ratio (cA/S) compared to hunger. The cA/S describes the narrowest opening at which it is still possible to walk through a door-like aperture without rotating the shoulders.

## Results

### Behavioral measurement of the body schema

**Figure 1:**
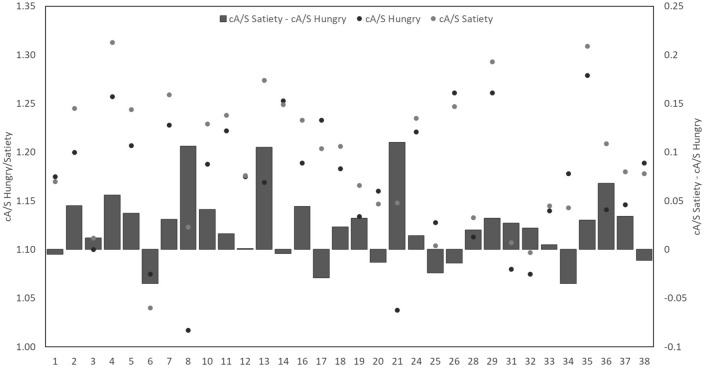
The subjects are plotted on the x-axis. On the left y-axis the absolute cA/S values are depicted for hungry (black circles) and satiated (grey circles). The subtraction of cA/S hungry from cA/S satiety is depicted on the right y-axis as bar chart. Positive values mean higher cA/S in the satiated condition compared to the hungry condition.

There was a significant difference in the cA/S between the hungry and satiated condition (t(31) = − 3.47, p < 0.005, d = 0.329, 95% CI [− 0.04, − 0.01]; Mhungry = 1.169, SD = 0.07; Msatiety = 1.192, SD = 0.07; Fig. 1), suggesting that satiated participants rotate their shoulder for relatively larger door widths compared to hungry .

### Hunger and satiety ratings

A repeated measures ANOVA with a Greenhouse–Geisser correction determined that the factor level of saturation (F(1, 31) = 5.90, p < 0.05. partial η^2^ = 0.160), as well as the factor time (F(1.79,55.62) = 4.09, p < 0.05. partial η^2^ = 0.117) showed a statistically significant difference between measurements. There was no significant interaction of the time * level of saturation (F(1.58,48.89) = 6.44, p = 0.06), (Hunger ratings: scalemean = 8.06, SD 1.24, meanbefore = 7.89 ± 1.14, meanmiddle = 8.20 ± 1.30, meanafter = 8.09 ± 1.63; satiety ratings: scalemean = 7.17, SD 1.66, meanbefore = 7.64 ± 1.67, meanmiddle = 7.23 ± 1.90, meanafter = 6.65 ± 1.95). However, a clear amount of hunger and satiety was reached at all measurement points.

### Correlation analyses

There were no significant correlations between the behavioural data and the BMI or the questionnaire Data (see Table 1).

**Table 1:** Correlation between the cA/S values for hungry and satiety and the clinical characteristics.

	cA/S hungry	cA/S satiety	cA/S_hungry − cA/S_satiety
BMI (kg/m^2^)	r = 0.062 p = 0.737	r = 0.137 p = 0.454	r = 0.135 p = 0.46
CES-D	r = − 0.52 p = 0.777	r = − 0.191 p = 0.295	r = − 0.249 p = 0.169
EDE-Q	r = 0.350 p = 0.05	r = 0.216 p = 0.236	r = − 0.236 p = 0.193
Restraint	r = 0.189 p = 0.3	r = 0.089 p = 0.628	r = − 0.177 p = 0.332
Weight concern	r = 0.284 p = 0.115	r = 0.191 p = 0.296	r = − 0.164 p = 0.370
Shape concern	r = 0.375 p = 0.034	r = 0.266 p = 0.142	r = − 0.193 p = 0.291
Eating concern	r = 0.136 p = 0.459	r = 0.016 p = 0.93	r = − 0.212 p = 0.244
FKB20			
Negative body evaluation	r = 0.294 p = 0.102	r = 0.213 p = 0.242	r = − 0.142 p = 0.438
Vital body dynamics	r = 0.126 p = 0.491	r = 0.187 p = 0.306	r = 0.109 p = 0.552
RSES			
Low self-esteem	r = 0.105 p = 0.568	r = 0.079 p = 0.669	r = − 0.046 p = 0.803
High self-esteem	r = − 0.114 p = 0.535	r = 0.01 p = 0.959	r = 0.22 p = 0.227
Mean hunger rating NRS	r = 0.152 p = 0.406		
Mean satiety rating NRS		r = − 0.080 p = 0.665	

*CES-D* the Center for Epidemiological Studies Depression Scale^28^, *EDE-Q* Eating Disorder Examination Questionnaire^29^, *FKB20* questions toward the body image^30^, *RSES* Rosenberg Self-esteem scale^31^, *NRS* numerical rating scale between 0 as neutral and 10 as very hungry resp. 0 as neutral and 10 as excessive full.

### Interrater reliability

The inter-rater reliability between the two evaluators was high (r = 0.99, p < 0.001).

## Discussion

The aim of this study was to investigate whether the body-scaled action can be influenced by the feeling of satiety. By means of a door like aperture we measured the cA/S, that a person perceives as too narrow to walk through and therefore rotates the shoulder. Our main result is a significantly higher cA/S if the participants were satiate compared to hungry, which indicates that satiated participants rotate their shoulder for relatively larger door widths compared to hungry. This suggests that satiety or hunger could modulate the body schema.

The analysis of the satiety ratings suggests that participants feeling of satiety decreased during the experiment. However, as the aperture width was randomized the influence on our results should be low.

The body schema is still a much-debated topic in the field of body awareness. A generally accepted definition still does not seem to exist. There also seem to be some aspects of the body schema that have not yet been explored. This study focuses on a short-term influence, assuming that the body schema has both short-term and long-term influences^5^.

Studies with body illusions showed short-term changes due to exteroceptive input such as visuospatial or proprioceptive stimuli. An example for a visuospatial stimulus is the Rubber-Hand-Illusion (RHI)^14,32^. For the induction of the RHI the real and the rubber hand are stroked synchronously. After the induction of the illusion subjects are asked to point to the position of the real hand with closed eyes. The mislocalisation between the real and the fake hand is called proprioceptive drift. Another example of a change in body schema is the extension of the body schema by the use of tools to reach distant objects^33–35^. The tool becomes part of the body and the environment which can be reached expands.

Pain is an example of an interoceptive influencing factor on the body schema. Although there are some studies that have shown that the emotional state^36^ or the feeling of satiety^25^ have an influence on the body image, there are to the best of our knowledge no similar studies for the body schema. Our study gives the first indication that somatic information such as the feeling of satiety can be an influencing factor on the body schema, too. The subjective feeling of hunger and the desire for food already changes significantly after only 1 day of over- or underfeeding^37^. This supports our finding that even a moderate rated feeling of hunger or satiety leads to a significant difference in movement.

The neural processing of hunger and satiety shows an overlap in the involved brain regions with the processing of body perception. A key region for both seems to be the IPL. During hunger, a complex network of the left striate and extrastriate cortex, IPL, and orbitofrontal cortex is active^38^, whereas saturation mainly activates the ventromedial prefrontal cortex, the dorsolateral prefrontal cortex and IPL^39,40^.

Body perception is associated with the extrastriate body area, the fusiform body area, and the right IPL^41–47^. The body schema is responsible for movement and interactions with the environment. It is anchored in the central nervous system^1^ and consists of two parts: a sensorimotor control network consisting of motor and somatosensory cortical regions, basal ganglia, thalamus and cerebellum, and a frontoparietal network that extends from the inferior frontal gyrus to the posterior parietal cortex (e.g. IPL)^41,48^. The former is responsible for the body representation and quick position-dependent corrections, the latter for the integration of afferent information, i.e. for the interaction with the environment.

Previous studies already used the cA/S as a measure to examine body perception and locomotion both, in obesity^49,50^ or patients with AN^51–55^, as well as in healthy participants^56–58^. In these studies, participants had to rate if they would fit to a pictured door^52,53^, walk through a door^57–60^, or walk through a door while a cover story is hiding the real aim of the study^51,54^. For example, in patients with AN the cA/S is significantly higher than in healthy participants^51–54^. This indicates both, a changed body image and a changed body schema in patients with AN.

Patients with AN or obesity also reported a changed perception of hunger and satiety^61^. For example, patients with AN reported a reduced feeling of hunger and a higher feeling of satiety than healthy participants^62^. They show a pronounced aversion to food stimuli^63,64^, while in healthy control subjects, especially when hungry, food stimuli are assessed positively^65,66^. It can therefore be assumed that the feeling of satiety in patients with AN could have an even greater impact on body perception than it is already the case in healthy participants.

Our study was able to demonstrate another interoceptive influencing factor on the body schema. Future studies have to evaluate how this knowledge might relate to eating disorders. Especially in AN, the feeling of hunger and the body schema are incongruent. The patients usually feel hungry, but move as if they were broader as they are. It would be interesting to investigate the effect of satiety on the body schema in AN. This knowledge might influence new approaches in body schema treatments.

## Methods

### Participants

Keizer et al.^54^ had an effect size of 1.21. As we investigated healthy controls in different states of satiety, we expected a lower effect size and used conservatively an effect size of 0.6 for the power calculation. For a within subject design and a paired sample t-test, an effect size of 0.6, an alpha error of 0.05 and a beta error of 0.95 a sample size of n = 32 is suggested. Thirty-eight healthy females (18–26 years) were recruited from advertisements at the university and surroundings in Bochum. Exclusion criteria were walking abnormalities, psychiatric diagnosis, a BMI outside the normal range of 18.5–24.9 kg/m^2^ and right assumptions regarding the real sense of the study. Additionally, psychology students in the third semester or higher were excluded to reduce the chance of revealing the cover story. After the experimental task the participants filled out demographic and clinical data (see Table 2), the Eating Disorder Examination Questionnaire ^EDE-Q^^29^ to exclude an eating disorder (Cut-off > 2.3), the Center for Epidemiological Studies Depression Scale ^CES-D^^67,28^ to exclude any depressed participants (Cut-off > 17), a questionnaire about body image^FKB-20^^30^, and about self-esteem^RSES^^31^, to better describe the sample.

**Table 2:** Demographic and clinical characteristics.

	M	SD	Range
**Demographic characteristics**			
Age (years)	22.28	1.938	19–26
BMI (kg/m^2^)	21.15	1.753	18.51–24.80
Shoulder width (cm)	39.03	2.521	34–44
**Clinical characteristics**			
CES-D	6.5	3.759	1.0–15.0
EDE-Q	0.787	0.664	0–2.29
EDE-Q restraint	0.781	0.943	0–3.2
EDE-Q weight concern	0.838	0.866	0–3.2
EDE-Q shape concern	1.173	0.913	0–3.88
EDE-Q eating concern	0.300	0.477	0–1.8
FKB20			
Negative body evaluation	14.063	3.918	8.0–22.0
Vital body dynamics	39.625	5.259	30–55
RSES			
Low self-esteem	10.063	3.301	5.0–16.0
High self-esteem	20.844	2.201	17.0–25.0

*CES-D* the Center for Epidemiological Studies Depression Scale^28^, *EDE-Q* Eating Disorder Examination Questionnaire^29^, *FKB20* questions toward the body image^30^, *RSES* Rosenberg Self-esteem scale^31^, *M* mean value, *SD* standard deviation.

Data from six participants were excluded due to instruction problems (n = 2), not fulfilling our cut-off-limitations in the questionnaires (n = 3) or understanding the real sense of the study (n = 1), this led to a final sample of n = 32. The mean age of the final sample was M = 22.28 years (SD = 1.94, range 19—26 years), mean body mass index (BMI) was M = 21.15 kg/m^2^ (SD = 1.75, range 18.51—24.8 kg/m^2^) and mean shoulder width was 39.03 cm (SD = 2.51, range 34—44 cm). Before the experiment all participants gave written informed consent and the experimental procedure was verbally explained by the researcher. The study was approved by the ethics committee and investigational review board of the faculty of medicine at the Ruhr University Bochum (approval number: 16-5916-BR) and adhered to the Declaration of Helsinki. Participation was compensated with 40 Euro.

### Experimental design

#### Experimental conditions

The experiment was conducted in our laboratory, Clinical and Experimental Behavioural Medicine, Department of Psychosomatic Medicine and Psychotherapy, LWL University Hospital, Ruhr University Bochum. Participants were invited for two experimental tests on separate days within 1 week to a similar time of the day. Each day had a different condition, hungry or satiated. The order of conditions was randomized using https://www.randomizer.org/. In advanced of the hungry condition, participants were instructed to dispense with food and beverages besides water and unsweetened tea at least for 12 h. The amount of hunger was rated on a numerical rating scale (NRS) between 0 as neutral and 10 as very hungry. Before the satiated condition the amount of satiety was rated on a NRS between 0 as neutral and 10 as excessive full. These ratings were conducted before each of the two parts (see below “Memory task”) of each condition and at the end of each measurement day. To provoke a comparable satiated feeling before the satiated condition the participants were asked to eat a 100 kcal snack and to drink at least 200 ml of water before the experiment and an additional glass of 200 ml water in the middle of the experiment.

#### Experimental procedure

Before the start of the experiment body-related measures like height, weight and shoulder width were assessed and five adhesive dots were affixed on the participants’ head, shoulders and feed. Participants were told that both are necessary to calibrate the cameras (see below “Rotation angle assessment and data analysis”). For the later analyses only the dots on the shoulder were used.

The experiment started by introducing the cover story (see below “Cover story”) and playing a listening comprehension story. Participants were asked to memorize (see below “Memory task”) as much as possible from the listening comprehension story to be able to answer questions related to this story. After 18 trials a second listening comprehension story was presented. In total 36 trials were completed in both conditions, consisting of 12 different aperture widths that each was presented three times in a randomized order (randomized using https://www.randomizer.org/). Aperture width was based on the actual shoulder width of the participants and ranged from an aperture-to-shoulder-ratio (A/S) = 0.9 to A/S = 1.45, in steps of A/S = 0.05. The aperture wide was changed while the experiment took place.

**Figure 2:**
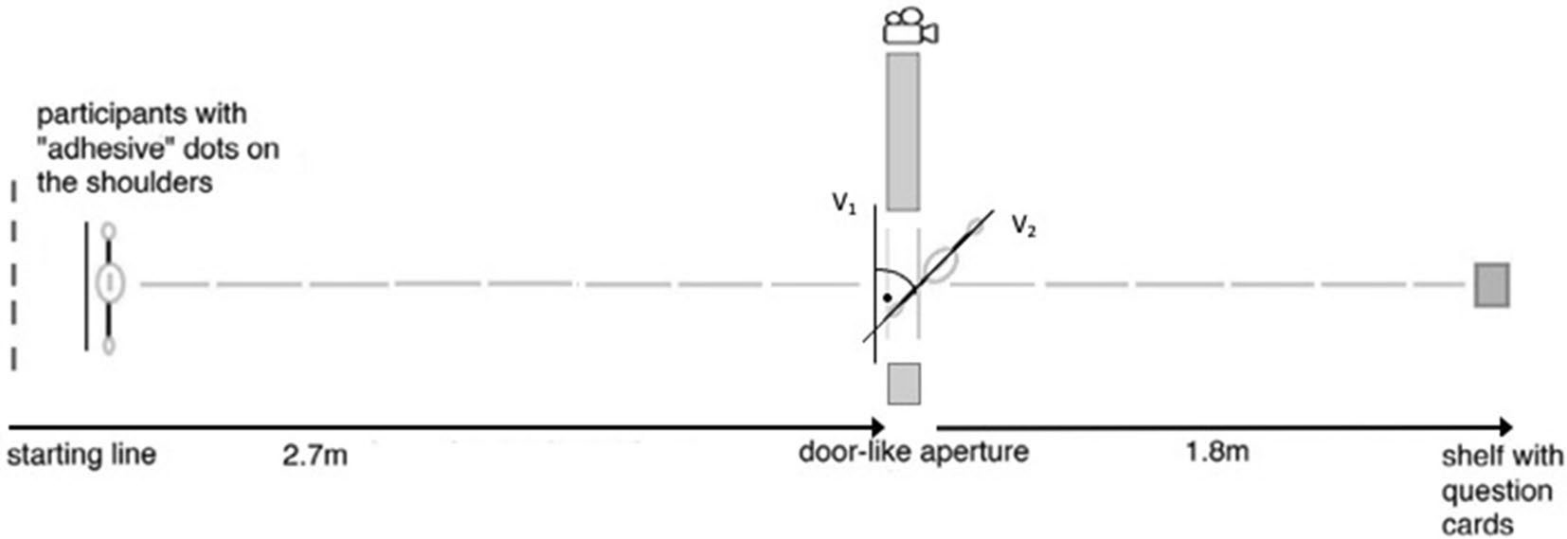
Schematic set up of the aperture and angle vectors to calculate shoulder rotation. Vector V2 points from the right to the left adhesive shoulder marker and vector V1 points from right to the left partition of the door aperture. Shoulder rotation was defined by calculating the angle between V1 and V2. An angle greater than 30° was defined as a rotation.

The experimental setup was a 4.5 m straight forward walking way. Each trial started on the starting line (see Fig. 2). Subsequently, participants were asked to walk a 4.5 m distance to the opposite end of the room, where a shelf with question cards was located. After 2.7 m they had to pass a door-like aperture, which consisted of a white painted pedestal (2.01 × 0.26 m) and a moveable white partition (1.97 × 1.23 m). To create an un-irritating atmosphere, white panels were chosen, and visual distractions were minimized. Participants crossed the door like aperture both on the way to the shelf and the way back to the starting line. Only the way to the shelf was used for the analyses.

After finishing the experimental procedure on the second day the participants had to fill in the questionnaires named above. Participants were asked about their opinion of the purpose of the study and were enlightened regarding the real purpose of the study afterwards.

#### Cover story

The study was announced to its participants as an experiment about ‘The Influence of the satiety feeling on the memory retention’ to be able to examine the unconscious part of the body experience.

To cover the real sense of the door crossings participants had to answer a memory question at multiple times throughout the experiment (see “Memory task”). They were told that the crossing of a door fosters forgetting. The reliability of this presented cover story was checked by asking participants about their opinion of the purpose of the study after the experiment. One question asked for the content of the story. The second question asked for the function of the camera. Just one participant suspected a conjunction between walking through the aperture, hunger or satiety, and body experience and was excluded.

#### Memory task

Both conditions, hunger and satiety, were divided into two parts. Both parts started with a short audio story. From four stories two stories per condition were chosen in a randomized order with a mean length of 5.6 min (range 4.25–6.4 min). Stories were not varying in difficulty, were all presented by the same male voice, and had each 12–14 questions associated. The participants were asked to listen to it and to remember as much details as possible.

After the first story participants were asked to walk from the starting line to the shelf at the back of the room, pick a card containing a question from the shelf, return to the starting line, read the question out loud and keep the answer in mind. Without yet answering they again walked through the aperture and returned to the starting point to answer the question. This was done to lead the focus to the memory tasks rather than onto the walking. This procedure was repeated 9 times. As only the way to the shelf was used for the analyses, this result in 18 door crossings for the analysis, with changes in the door width in randomized order based on the individual A/S-ratio. A change in door width was made every time before participants started their way towards the shelf.

For the remaining 9 trials consisting of 18 door crossings a new listening comprehension story was presented. It was announced, that the investigation was now focused on the short term memory. After crossing the door and taking a card, participants now had to read the question immediately before walking back to the starting point. Back at the starting point, they were asked to recite the question freely. Answering the question and crossing the door aperture was performed as before.

### Rotation angle assessment and data analysis

Throughout the experiment a Logitech c920 HD Pro webcam camera (Logitech, Lausanne, Switzerland) hanging on the ceiling recorded the test persons’ movements. The camera filmed the door-aperture from right above and was used to evaluate rotation vectors. The videos were recorded with ManyCam 5 ©2006–2016 Visicom Media Inc.. Due to technical problems with this program nine trails of one condition for five participants could not be analysed.

After the experiment, from the videos of each of the 36 door crossings a snapshot was saved that recorded the moment participants were crossing the door-aperture. Serving as reference, two lines drawn to the ground were taken as reference points. The adhesive dot on the shoulder of each participant had to be between these lines as the snapshot was taken. Shoulder rotations were measured in these photos using MB-Ruler (5.3 ©Markus Bader- MB-Softwaresolutions, Iffezheim, Germany). The line connecting both parts of the door arch (V1) and the line between the two adhesive dots on the participants’ shoulders (V2) were taken to measure the angle vector (see Fig. 2). An angle above 30° was defined as a rotation.

Subsequently every existing angle of one condition was added in a coordinate system, in which the angle minus 30° formed the y-axis and the Aperture-to-Shoulder-ratio formed the x-axis. A second-degree polynomial regression analysis was calculated, and the intercept of this function and the y-axis yielded the cA/S. This was done for each participant on both conditions. The cA/S of both conditions were compared with a paired-sampled t-test after testing for normal distribution.

The measurement time points (factor time: before, in the middle, and after the experiment) of the hunger and satiety ratings (factor level of saturation) were compared with a two-factor ANOVA with repeated measures for hunger and satiety.

Furthermore, we performed a correlation analysis using Pearsons' coefficient (r) to test the interconnection between clinical data and behavioural measures. To verify the reliability of the rotation angle assessment, the analysis was carried out by a second researcher. The interrater reliability of the cA/S was investigated by Pearson correlation.

The level of significance was set at p < 0.05. Cohen's d effect size and 95% confidence interval are reported. Statistical analysis was conducted with IBM® SPSS® Statistics 26 (Chicago, IL, USA).

## Data Availability

All data presented in this article can be accessed via https://osf.io/dxw94/.
